# Discrimination of *Curcuma* species from Asia using intron length polymorphism markers in genes encoding diketide-CoA synthase and curcumin synthase

**DOI:** 10.1007/s11418-021-01558-2

**Published:** 2021-09-05

**Authors:** Qundong Liu, Shu Zhu, Shigeki Hayashi, Osamu Iida, Akihito Takano, Katsunori Miyake, Suchada Sukrong, Mangestuti Agil, Indira Balachandran, Norio Nakamura, Nobuo Kawahara, Katsuko Komatsu

**Affiliations:** 1 Institute of Natural Medicine, University of Toyama, 2630, Sugitani, Toyama 930-0194 Japan; 2 Research Center for Medicinal Plant Resources, National Institutes of Biomedical Innovation, Health and Nutrition, Kumage-Gun, 17007-2 Nakatane-cho, Kagoshima, 891-3604 Japan; 3 Showa Pharmaceutical University, 3-3165 Higashi-Tamagawagakuen, Machidashi, Tokyo 194-8543 Japan; 4 Tokyo University of Pharmacy and Life Sciences, 1432-1 Horinouchi, Hachioji, Tokyo 192-0392 Japan; 5 Chulalongkorn University, 254 Phayathai Rd, Wang Mai, Pathum Wan District, Bangkok, 10330 Thailand; 6 Airlangga University, Jl. Airlangga No.4 - 6, Airlangga, Kec. Gubeng, Kota SBY, Jawa Timur 60115 Indonesia; 7 Center for Medicinal Plants Research, Arya Vaidya Sala, Kottakkal, Malappuram, Kerala 676503 India; 8 Doshisha Women’s College of Liberal Arts, Kodo, Kyotanabe City, Kyoto 610-0395 Japan

**Keywords:** *Curcuma*, Intron length polymorphism, Diketide-CoA synthase, Curcumin synthase, *trn*K, Molecular identification

## Abstract

Recently, *Curcuma* rhizome-related foods with claimed health benefits have been used worldwide; however, correct identification and quality assessment have not been conducted. Due to the wide distribution and morphological similarities of *Curcuma* species, the classification of some species is debated and nomenclature is inconsistent among countries. In this study, to elucidate specific molecular markers of medicinally used *Curcuma* species in Asia, and to solve the confusion on the reported botanical origin of crude drugs, molecular analysis based on the intron length polymorphism (ILP) in genes encoding diketide-CoA synthase and curcumin synthase and the *trn*K intron sequences was performed using 59 plant specimens and 42 crude drug samples from 13 *Curcuma* species, obtained from Asian countries. The ILP patterns of the respective species from both plant specimens and crude drug samples revealed high consistency in *C. aromatica*, *C. zedoaria*, *C. phaeocaulis*, *C. aeruginosa*, *C. wenyujin,* and *C. zanthorrhiza*, but showed intraspecies polymorphism in *C. longa*, *C. kwangsiensis*, *C. amada*, *C. mangga* and *C. comosa*. The *C. longa* specimens and samples were separated into three subgroups which were highly consistent with their geographical origins. Based on the ILP markers and the *trn*K intron sequences, the botanical origins of “Khamin oi” from Thailand were correctly determined to be *C. longa* or a hybrid between *C. longa* and other species, and “Wan narn kum” from Thailand and “Kasturi manjal” from India were correctly determined to be *C. zanthorrhiza*.

**Supplementary Information:**

The online version contains supplementary material available at 10.1007/s11418-021-01558-2.

## Introduction

Genus *Curcuma* (Zingiberaceae) comprises approximately 120 species that grow widely in subtropical and tropical Asia. The most widely distributed and economically valuable species *C. longa* L. is cultivated around the world. The dried rhizome of *C. longa*—called turmeric in English, “Ukon” in Japanese, “Jianghuang” in Chinese, “Haldi” in Hindi, “Khamin chan” in Thai and “Kunyir” in Javanese—has been used as a traditional crude drug, spice, dye, cosmetic as well as a health food in Asian countries. A number of other *Curcuma* species are cultivated and used in Asia: *C. phaeocaulis* Valeton, *C. kwangsiensis* S. G. Lee et C. F. Liang and *C. wenyujin* Y. H. Chen et C. Ling cultivated in China as the traditional Chinese crude drug “Ezhu”; *C. zedoaria* (Christm.) Roscoe and *C. aromatica* Salisb. cultivated in Japan as the crude drug “Gajyutsu” and the health food “Haru-ukon,” respectively; *C. zedoaria*, *C. aromatica*, *C. zanthorrhiza* Roxb., *C. aeruginosa* Roxb., *C. caesia* Roxb. and *C. amada* Roxb. in India as Ayurveda crude drugs, folk medicine or a source of starch; *C. zedoaria*, *C. aromatica*, *C. comosa* Roxb., *C. aeruginosa* and *C. mangga* Valeton et Zijp in Thailand as crude drugs or foods; *C. zanthorrhiza*, *C. zedoaria*, *C. aeruginosa* and *C. heyneana* Valeton et Zijp in Indonesia as crude drugs or a source of starch [1–4]. The medicinal properties of *C. longa* are mainly attributed to its abundant content of curcuminoids which have been reported to possess anti-inflammatory, antioxidant and anticancer activities [5–7]. However, other *Curcuma* drugs that contain no or few curcuminoids but characteristic essential oils also have pharmacological effects. For example, *C. phaeocaulis* rhizome showed anti-inflammatory activity [8] and cyclooxygenase-2 inhibitory activity in vitro, with furanodienone and curcumenol identified as the active constituents [9].

Recently, with the increasing popularity of foods with health claims and so-called “health food,” including those derived from *Curcuma* rhizomes in Japan and other countries, the *Curcuma* rhizomes mentioned above are frequently used worldwide; however, correct identification and quality assessment have not been performed. Due to the wide distribution and morphological similarities of *Curcuma* species, the classification of some species is debated and nomenclature is inconsistent among countries, especially for *C. aromatica* and *C. zedoaria*. This situation leads to confusion in the use of *Curcuma* crude drugs. Multiple DNA regions have been tested for their capability in discrimination of *Curcuma* species, including *mat*K, *rbc*L, *rpo*C1, *rpo*B, *rps*36-*rps*8, *ndh*J, *trn*L-F, *trn*H-*psb*A, *acc*D and *trn*S-*trnf*M of chloroplast DNA [10–13] as well as internal transcribed spacer (ITS) regions of nuclear DNA [10, 13]. However, these chloroplast DNA regions revealed limited resolution due to the high conservation in their sequences [10–13]. The ITS sequences showed high polymorphism even within a single individual, thus a cloning method is required for further analyses [10, 13]. We performed molecular analysis on the *trn*K intron region of chloroplast DNA to discriminate six *Curcuma* species from China and Japan, and detected five main sequence types [14, 15]. Moreover, by using a new marker based on the intron length polymorphism (ILP) of genes encoding diketide-CoA synthase (DCS) and curcumin synthase (CURS), the two important enzymes involved in the biosynthesis of curcuminoids, there were distinguishable ILP patterns of *C. longa*, *C. phaeocaulis*, *C. zedoaria*, *C. kwangsiensis*, *C. aromatica* and *C. wenyujin* as well as intraspecies variation of *C. longa* and *C. kwangsiensis* [16]. The ILP markers, including PCR amplicons of two intron regions in the two DCS genes and one intron in the three CURS genes (Fig. S1), showed potential for discrimination of *Curcuma* plants and related products. However, the tested specimens and crude drug samples were mostly limited to those from China and Japan. Further study with a large sample size including *Curcuma* species and *Curcuma*-related crude drugs from Southeast and South Asia, such as Thailand, Indonesia and India is needed. The present study aims to elucidate specific ILP patterns of medicinally used *Curcuma* species in Asia, to locate the original habitats of some *Curcuma* species cultivated in Japan, and to resolve the confusion caused by inconsistent scientific names among countries, especially those reported as the botanical origin of crude drugs. To do this, molecular analysis based on the ILP markers of *DCS* and *CURS* genes and the *trn*K intron sequences was performed using a number of *Curcuma* specimens and crude drug samples obtained from Japan, China, Thailand, Indonesia, India, and other Asian countries.

## Materials

Fifty-nine plant specimens of 11 *Curcuma* species including *C. longa*, *C. aromatica*, *C. phaeocaulis*, *C. aeruginosa*, *C. zedoaria*, *C. zanthorrhiza*, *C. wenyujin*, *C. kwangsiensis*, *C. amada*, *C. petiolata* Roxb. and *C. sichuanensis* X. X. Chen, and uncertain species such as *C. mangga* were mainly collected from several medicinal plant gardens in Japan (Table 1). Most of them were introduced from China, Thailand, Indonesia, India, Malaysia and Nepal. Forty-two crude drug samples were widely collected from various markets in Thailand, India, Indonesia, China, Japan, Myanmar, Nepal and Sri Lanka (Table 2). The vouchers were deposited in the Museum of Materia Medica, Institute of Natural Medicine, University of Toyama (TMPW). Botanical origins of crude drug samples (Table 2) were deduced from their local names by referring to the literature [3, 4, 17–19].

**Table 1:** Plant specimens

Scientific name*	Voucher no.	Collected from	Original habitat	Local name	Collection date
*C. longa*	Q-32	T-RCMPR^a^	Nanning, Guangxi, China	Huangjiang	1999.7
*C. longa*	Q-33	T-RCMPR	Jiangjin, Chongqing, China	Jianghuang	1999.8
*C. longa*	Q-72	T-RCMPR	Leshan, Sichuan, China	Huangjiang	2000.8
*C. longa*	Ko-92	T-RCMPR	Tanegashima, Japan	Ukon	2019.7
*C. longa*	00003	SPU^b^	Okinawa, Japan	** − **	2000.1
*C. longa*	10162	TUPLS^c^	Japan	Ukon	1980
*C. longa*	Me-1	T-RCMPR	Japan	Ukon	2019.7
*C. longa*	Q-62	T-RCMPR	Chiong Mai, Thailand	Kamin-chan	2001.3
*Curcuma* sp. (*C. longa*)	94009	SPU	Thailand	** − **	1994.4
*C. longa*	T002	TMPW^d^	Chiong Mai, Thailand	Kamin-chan	2011.11
*C. longa*	T004	TMPW	Wang Nam Yen, Thailand	Kamin-chan	2011.11
*C. longa*	No-16	T-RCMPR	Indonesia	Ukon	2019.7
*C. longa*	Tsu-7	T-RCMPR	Java, Indonesia	Ukon	2019.7
*Curcuma* sp. (*C. longa*)	97021	SPU	Iduli Village, India	** − **	1997.7
*C. longa*	I-0010	TMPW	Arya Vaidya Sala, Kottakkal, Kerala, India	** − **	2000.12
*C. longa*	92005	SPU	Kathmandu, Nepal	** − **	1992.2
*C. aromatica* (Jp)	01005	SPU	T-RCMPR, Tanegashima, Japan	** − **	2001.4
*C. aromatica* (Jp)	10164	TUPLS	Japan	Haru-ukon	1981–1985
*C. aromatica* (Jp)	K-116	T-RCMPR	Japan	Haru-ukon	2019.7
*C. aromatica* (Jp)	H-21	T-RCMPR	Japan	Haru-ukon	2019.7
*C. aromatica* (Jp)	Me-3	T-RCMPR	Japan	Haru-ukon	2019.7
*C. aromatica* (Jp)	J-12	T-RCMPR	Japan	Haru-ukon	2019.7
*C. aromatica* (Cn)	Q-35	T-RCMPR	MBGB^e^, Beijing, China	Yujin	1999.7
*C. aromatica* (Cn)	Q-36	T-RCMPR	GG^f^, Guangzhou, Guangdong, China	Mao-yujin	1999.7
*C. aromatica* (Cn)	Q-37	T-RCMPR	GMPG^g^, Nanning, Guangxi, China	Yujin	1999.7
*Curcuma* sp. (*C. aromatica*)	94006	SPU	Thailand	** − **	1994.4
*C. phaeocaulis*	Q-38	T-RCMPR	GMPG, Nanning, Guangxi, China	Peng-ezhu	1998.9
*C. phaeocaulis*	Q-39	T-RCMPR	GMPG, Nanning, Guangxi, China	Heixinjiang	1999.7
*C. phaeocaulis*	Q-40	T-RCMPR	KBG^h^, Kunming, Yunnan, China	Heihe-jianghuang	1999.8
*C. phaeocaulis*	Q-42	T-RCMPR	CACMM^i^, Chongqing, China	Peng-ezhu	1999.8
*C. phaeocaulis*	Q-43	T-RCMPR	Jingxi, Guangxi, China	Peng-ezhu	1999.8
*C. phaeocaulis*	Q-64	T-RCMPR	Longwan, Guangdong, China	Ezhu	2001.9
*Curcuma* sp. (*C. phaeocaulis*)	94010	SPU	Thailand	** − **	1994.4
*C. aeruginosa*	Q-41	T-RCMPR	XTBG^j^, Menglun, Yunnan, China	Peng-ezhu	1999.8
*C. aeruginosa*	Q-47	T-RCMPR	XTBG, Menglun, Yunnan, China	Ezhu	1999.8
*C. aeruginosa*	10167	TUPLS	Indonesia		1980
*C. zedoaria* (Jp)	91014	SPU	TMMPG^k^, Tokyo, Japan	** − **	1991.6
*C. zedoaria* (Jp)	K-100	T-RCMPR	T-RCMPR, Tanegashima, Japan	Gajyutsu	2019.7
*C. zedoaria* (Jp)	Ko-93	T-RCMPR	T-RCMPR, Tanegashima, Japan	Gajyutsu	2019.7
*C. zedoaria* (Ind)	I-0005	TMPW	Arya Vaidya Sala, Kottakkal, Kerala, India	** − **	2000.12
*C. zanthorrhiza*	Q-48	T-RCMPR	Xishuangbanna, Yunnan, China	Huanghong-jianghuang	1999.8
*C. zanthorrhiza*	K-105	T-RCMPR	Japan	Kusuri-ukon	2019.7
*C. zanthorrhiza*	10163	TUPLS	Malaysia	Kusuri-ukon	1995
*C. zanthorrhiza*	94020	SPU	Denpasar, Bali, Indonesia	** − **	1994.4
*C. zanthorrhiza*	I-0009	TMPW	Arya Vaidya Sala, Kottakkal, Kerala, India	** − **	2000.12
*C. wenyujin*	GZ55-2	TMPW	Ruian, Zhejiang, China	Pian-jianghuang	2001.9
*C. wenyujin*	Q-49	T-RCMPR	GMPG, Nanning, Guangxi, China	Wen-yujin	1999.7
*C. wenyujin*	Q-70	T-RCMPR	Ruian, Zhejiang, China	Pian-jianghuang	2001.9
*C. kwangsiensis*	Q-63	T-RCMPR	Longwan, Guangdong, China	Ezhu	2001.9
*C. kwangsiensis*	Q-66	T-RCMPR	Dapingshan, Guangxi, China	Ezhu	2001.9
*C. kwangsiensis*	Q-67	T-RCMPR	Dapingshan, Guangxi, China	Ezhu	2001.9
*C. kwangsiensis*	Q-68	T-RCMPR	Luwu, Guangxi, China	Ezhu	2001.9
*C. kwangsiensis*	Q-69	T-RCMPR	Shangsi, Guangxi, China	Ezhu	2001.9
*C. amada*	I-0007	TMPW	Arya Vaidya Sala, Kottakkal, Kerala, India	** − **	2000.12
*Curcuma* sp. (*C. amada*)	00591	TUPLS	India	Mango-gajyutsu	2016
*Curcuma* sp. (*C. mangga*)	00959	TUPLS	Thailand	Kamin-kao	2018
*C. petiolata*	K-2	TMPW	Kyoto, Japan	Hana-ukon	2019.3
*Curcuma* sp. (*C. petiolata*)	94008	SPU	Thailand	** − **	1994.4
*C. sichuanensis*	Q-50	T-RCMPR	Chongqing, China	Chuan-yujin	1998.4

^a^T-RCMPR: Tanegashima branch, Research Center for Medicinal Plant Resources, National Institutes of Biomedical Innovation, Health and Nutrition, Japan

^b^SPU: Showa Pharmaceutical University, Tokyo, Japan

^c^TUPLS: Tokyo University of Pharmacy and Life Sciences, Tokyo, Japan

^d^TMPW: Museum of Materia Medica, Institute of Natural Medicine, University of Toyama

^e^MBGB: Medicinal Plant Garden of Institute of Chinese Materia Medica, China Academy of Chinese Medical Sciences

^f^GG: Ginger Garden of South China Institute of Botany, Academia Sinica

^g^GMPG: Guangxi Medicinal Plant Garden

^h^KBG: Kunming Botany Garden

^i^CACMM: Herbal Garden of Chongqing Academic of Chinese Materia Medica

^j^XTBG: Xishuangbanna Tropical Botanical Garden, Academia Sinica, k) TMMPG: Tokyo Metropolitan Medicinal Plant Garden

** − ** Data unavailable

*Scientific names of *C. aromatica* and *C. zedoaria* are followed by the name of each country where the plant was produced: (Jp), Japan; (Cn), China; (Ind), India

**Table 2:** Crude drug samples

Producing area	TMPW no.	Local name	Species^a^*	Market	Collection date	*trn*K type^c^	ILP group^d^
Thailand	D20992	Khamin chan	*C. longa*	Chiang Mai, Thailand	2001.11	Ltk(10T)	**L1**
	D24869	Khamin chan	*C. longa*	Damnoen Saduak, Ratchaburi, Thailand	2005.11	Ltk(11T)in-1	**L2**
	D24887	Khamin chan	*C. longa*	Bangkok, Thailand	2005.11	Ltk(11T)in-2	**L2**
	D24864	Khamin oi	*C. zedoaria* (Thai)	Damnoen Saduak, Ratchaburi, Thailand	2005.11	Ltk(11T)	**L1**
	D21642	Khamin oi	*C. zedoaria* (Thai)	Damnoen Saduak, Ratchaburi, Thailand	2002.11	K(pl)Ztk(7A13T)	**L2**
	D24876	Khamin oi	*C. zedoaria* (Thai)	Bangkok, Thailand	2005.11	K(pl)Ztk(7A13T)	**L2**
	D22069	Wan maha mek	*C. aeruginosa*	Bangkok, Thailand	2002.11	Ptk	**P2**
	D30522	Wan maha mek	*C. aeruginosa*	Bangkok, Thailand	2019.9	K(pl)Ztk(7A15T)	**C**
	D30517	Wan chak modluk	*C. comosa*	Bangkok, Thailand	2019.9	K(pl)Ztk(7A15T)	**C**
	D30519	Wan chak modluk	*C. comosa*	Bangkok, Thailand	2019.9	K(pl)Ztk(7A13T)	**C**
	D30521	Wan chak modluk	*C. comosa*	Bangkok, Thailand	2019.9	K(pl)Ztk(7A15T)	**C**
	D30523	Wan chak modluk	*C. comosa*	Bangkok, Thailand	2019.9	K(pl)Ztk(7A15T)	**C**
	D24866	Wan chak modluk	*C. comosa*	Damnoen Saduak, Ratchaburi,Thailand	2005.11	K(pl)Ztk(7A15T)	**C**
	D24867	Wan narn kum	*C. aromatica* (Thai)	Damnoen Saduak, Ratchaburi, Thailand	2005.11	K(pl)Ztk(6A14T)	**Za**
India	D20480	Manjal	*C. longa* [Allepy]	Trivandrum, India	2000.12	Ltk(11T)	**L3**
	D20478	Manjal	*C. longa* [Tamil Nadu]	Cochin, India	2000.12	Ltk(11T)	**L3**
	D20494	Pasappu	*C. longa* [Nizamabad]	Nizamabad, India	2000.12	Ltk(11T)	**L3**
	D20486	Pasappu	*C. longa* [Guntur]	Hyderabad, India	2000.12	Ltk(11T)	**L3**
	D24455	Ukon^b^	*C. longa*	Tokyo, Japan	2005.4	Ltk(11T)	**L3**
	D20477	Kasturi manjal	*C. aromatica* (Ind)	Cochin, India	2000.12	K(pl)Ztk(6A14T)	**Za**
	D20483	Kasturi manjal	*C. aromatica* (Ind)	Trivandrum, India	2000.12	K(pl)Ztk(6A14T)	**Za**
	D22836	Kastrui manjal	*C. aromatica*	Madras, India	2004.11	K(pl)Ztk(7A13T)	**Za**
Indonesia	D24984	Kunir	*C. longa*	Yogyakarta, Indonesia	2006.3	Ltk(11T)	**L3**
	D25843	Kunir putih	*C. zedoaria* (Inn)	Bogor, Indonesia	2008.5	K(pl)Ztk(6A14T)	**Ze**
	D24986	Tamu ireng	*C. aeruginosa*	Yogyakarta, Indonesia	2006.3	Ptk	**Ze**
	D14119	Temu lawak	*C. zanthorrhiza*	Semarang, Indonesia	1994.2	K(pl)Ztk(6A14T)	**Za**
	D14151	Temu lawak	*C. zanthorrhiza*	Tawangmangu, Indonesia	1994.2	K(pl)Ztk(6A14T)	**Za**
	D24987	Temu lawak	*C. zanthorrhiza*	Yogyakarta, Indonesia	2006.3	K(pl)Ztk(6A14T)	**Za**
	D25841	Temu lawak	*C. zanthorrhiza*	Bogor, Indonesia	2008.5	K(pl)Ztk(6A14T)	**Za**
	D24983	Kunir manga	*C. mangga*	Yogyakarta, Indonesia	2006.3	Ptk	**A/M**
	D27151	Temu manga	*C. mangga*	Surabaya, Indonesia	2010.9	Ptk	**A/M**
	D27130	Temu manga	*C. mangga*	Solo, Indonesia	2010.9	Ptk	**A/M**
China	D20208	Jianghuang	*C. longa*	Sichuan, China	2000.8	Ltk(10T)	**L1**
	D29970	Ukon^b)^	*C. longa*	Osaka, Japan	2018.9	Ltk(11T)	**L1**
	D20237	Wenzhu	*C. phaeocaulis*	Sichuan, China	2000.8	Ptk	**P1**
	D22297	Gajyutsu^b)^	*C. phaeocaulis*	Osaka, Japan	2003.10	Ptk	**P1**
Japan	D20285	Gajyutsu	*C. zedoaria* (Jp)	Osaka, Japan	2001.6	K(pl)Ztk(6A14T)	**Ze**
	D25344	Murasaki ukon	*C. zedoaria* (Jp)	Osaka, Japan	2007.4	K(pl)Ztk(6A14T)	**Ze**
Myanmar	D30516	Thayetkin	*C. amada*	Yangon, Myanmar	2016.12	Ptk	**A/M**
	D30515	Thayetkin	*C. amada*	Dawei, Myanmar	2018.11	Ptk	**A/M**
Nepal	D8535	Hharedo	*C. longa*	Kathmandu, Nepal	1963.11	Ltk(10T)	**L1**
Sri Lanka	D8685	Kaha	*C. longa*	Anuradhapura, Sri Lanka	1980.1	Ltk(11T)139A	**L3**

^a^Botanical origin was deduced from local name of crude drug

^b^Name of crude drug in Japanese market

^c^Result according to *trn*K intron sequencing

^d^Grouping result according to the analyzed ILP patterns

*Scientific names of *C. aromatica* and *C. zedoaria* are followed by the name of each country where the plant was produced: (Thai), Thailand; (Ind), India; (Inn), Indonesia; (Jp), Japan. For Indian *C. longa*, varieties name are added in parentheses

## Methods

### Morphology of plant specimens

With regard to plant specimens obtained from medicinal plant gardens, their morphological features including the internal color of rhizomes, color of the leaf sheath, presence or absence of a purple band on and around the leaf midvein, the presence or absence of hairs on upper and lower sides of leaves, the position of inflorescence and the color of bracts in terminal and lower parts of the inflorescence were compared with botanic literatures [3, 18–21] for morphological identification. Those already preserved in the TMPW museum were identified by Dr. Indira Balachandran, Center for Medicinal Plants Research, Arya Vaidya Sala, India, and Dr. Katsuko Komatsu, Institute of Natural Medicine, University of Toyama, Japan.

### Isolation of total DNA

Total DNA was extracted from 40 to 50 mg of dried leaves of plant specimens or 80–100 mg of dried rhizomes of crude drug samples using a DNeasy Plant Mini Kit (Qiagen, Germany) according to the manufacturer’s instructions with some modifications [22]. A 1-μL aliquot of extraction solution of each sample was examined using 1.0% agarose gel electrophoresis stained with UltraPower DNA Safedye (Gellex International, Japan) to check the condition of total DNA.

### PCR amplification and sequence analysis of *trn*K intron regions

The *trn*K intron regions of plant specimens and crude drug samples were amplified via PCR using two pairs of primers [14, 15]: trnK3914F (5′-TGG GTT GCT AAC TCA ATG G-3′) and CT911R (5′-TAT AGA AAG TGT TGT TGC CG-3′) for upstream intron regions; and CT2240F (5′-TTG CAA AGA TTA AGT TCG GG-3′) and trnK2R (5′-AAC TAG TCG GAT GGA GTA G-3′) for the downstream intron regions (Fig. S2). Of PCR solution, 25 μL contained 10–100 ng of total DNA as a template, 1 × Buffer for KOD-Plus, 0.2 mM dNTPs, 1.0 mM MgSO_4_, 0.4 μM of each primer and 0.5 U of KOD-Plus polymerase (Toyobo, Japan). The PCR amplification was carried out with a Takara PCR Thermal Cycler TP600/650 (Takara, Japan). The cycling condition was a hot start at 94 °C for 4 min, followed by 35 cycles of denaturation at 94 °C for 30 s, annealing at 50 °C for 30 s, extension at 68 °C for 60 s and a final extension at 68 °C for 7 min. For samples for which this PCR amplification failed, the KOD-FX Neo DNA polymerase system (Toyobo) was then used, in which 25 μL of solution consisted of 12.5 μL of 2 × Buffer for KOD-FX Neo, 0.2 mM dNTPs, 0.4 μM of each primer and 0.5 U of KOD-FX Neo polymerase. The PCR cycling condition was the same as that of the KOD-Plus system. A 1-μL PCR product of each sample was examined using 1.0% agarose gel electrophoresis. Then, the PCR products were purified using Wizard SV Gel and PCR Clean-Up System (Promega, USA).

The sequencing reaction was performed with 50 ng of each purified PCR product as a template and each of the primers CT23F (5′-AGT ACT CGG CTT TTA AGT GC-3′) or CT828R (5′-TGA AGC AGA GGT AGA AGG AAC-3′) for the upstream intron region and each of CT2240F or CT2675R (5′-TTT TCC TTG TTA TAA TAG GT-3′) for the downstream intron region [15, 16]. The 10 μL sequencing reaction mixture contained 1.8 μL of BigDye Sequencing Buffer (ThermoFisher, USA), 0.5 μL of BigDye Terminator v3.1 (ThermoFisher) and 0.35 μM primer. The cycling condition used for the sequencing reaction was a hot start at 96 °C for 1 min, followed by 26 cycles of denaturation at 96 ℃ for 10 s, annealing at 50 °C for 5 s and extension at 60 °C for 4 min. The sequencing reaction products were purified using BigDye XTerminatorTM Purification Kit (ThermoFisher), then sequences of the respective purified products were determined by an ABI Prism 3100-Avant Genetic Analyzer (ThermoFisher). Sequencing data were collected with 3100-Avant Data Collection Software (v5.3, ThermoFisher) and sequences were assembled with Sequencing Analysis Software (v5.3, ThermoFisher). Consensus sequences were aligned and compared using Multalin software (http://multalin.toulouse.inra.fr/multalin/) or BioEdit (ver. 4.0.6.2). The determined *trn*K intron sequences were registered in the International Sequence Database (INSD: DDBJ/EMBL/GenBank) with the accession numbers shown in Table 4.

### PCR amplification and size determination of the amplicons of intron regions of *DCS1*, *DCS2* and *CURS1–CURS3*

Two intron regions I and II in *DCS1* and *DCS2* and one intron region in *CURS1–CURS3* were amplified separately via PCR using each of the three pairs of primers [16]: DCSI-F (5′-GAC TWC TAY TTC CGS GTC AC-3′) and DCSI-R (5′-GAG CCA GCA ARC TMG GAT TC-3′); DCSII-F (5′-CCA CAT CGA GAG CCT CTT CG-3′) and DCSII-R (5′-CTG GCT YTT SAG GTG GAA GGT C-3′); and CURS-F (5′-GAC TWC TAY TTC CGS GTC AC-3′) and CURS-R (5′-CTT SGG CCK CTS CTT CAG GAT C-3′). Primers DCSI-F, DCSII-R and CURS-R were labeled with fluorescent dyes 6-FAM, HEX and CY-3, respectively, which enabled the respective amplicons to be detected and discriminated. A KOD-Plus or KOD-FX Neo DNA Polymerase Kit was used for PCR amplification and the composition of the common ingredients in PCR solution was the same as that described in the section “PCR amplification and sequence analysis of *trn*K intron regions”. The cycling condition was a hot start at 98 °C for 4 min, followed by 35 cycles of denaturation at 98 °C for 10 s, annealing at 58 °C for 30 s, extension at 68 °C for 60 s and a final extension at 68 °C for 7 min. For each sample, 1 μL of PCR product was examined using 1.0% agarose gel electrophoresis. Successfully amplified fragments were diluted with dH_2_O in ratio range of 10–50. Then, 0.5 μL of diluted PCR product of each intron region was mixed with 9.0 μL of Hi-Di Formamide (ThermoFisher) and 0.5 μL of GeneScan 600 LIZ Size Standard (ThermoFisher). The size of the amplified fragments was determined by an ABI Prism 3100-Avant Genetic Analyzer (ThermoFisher) with GeneMapper software (ver. 3.5, ThermoFisher). Determining the fragment length in ILP analysis followed two steps: compared with size standard markers that included 36 single-stranded labeled fragments ranging within 20–600 bp; fragment length was calculated automatically and the length value was manually adjusted within ± 1 bp by rounding and comparing with reference samples of the respective species.

### Dendrogram analysis

The fragment pattern in the three intron regions of each sample was converted to a binary character string by scoring each fragment as 1 (presence) or 0 (absent), and then a proportional distance matrix was generated using the PAST software (ver. 4.0). Cluster analysis was conducted and a dendrogram was constructed based on the matrix by neighbor-joining (NJ) method [23] using the MEGA X software (ver. 10.1.6) [24].

## Results and discussion

### Morphological identification of plant specimens

Fifty-two plant specimens were morphologically identified according to taxonomic literature [3, 18–21]. The informative morphological features for discrimination of *Curcuma* species including information on rhizomes, leaves and inflorescences were observed and summarized (Table 3).

**Table 3:** Morphological characters of plant specimens

Scientific name*	Voucher no.	Original habitat	Morphological characters							
			Rhizome	Leaf sheath	Leaf blade			Inflorescence		
			Color of inside	Color	Purple band	Hair on surface		Position	Color of bract	
					(Shape)	Upper	Lower		Terminal part	Lower part
*C. longa*	Q-32	China	Bright yellow	Green	** − **	** − **	** − **	Central	White & pink	Pale green
*C. longa*	Q-33	China	Bright yellow	Green	** − **	** − **	** − **	Central	Red	Pale green & red
*C. longa*	Q-72	China	Bright yellow	Green	** − **	** − **	** − **	Central	Red	Pale green & red
*C. longa*	Ko-92	Japan	Orange	Green	** − **	** − **	** − **	Central	White & pink	Pale green
*C. longa*	00,003	Japan	Bright yellow	Green	** − **	** − **	** − **	**/**	**/**	**/**
*C. longa*	10,162	Japan	Orange	Green	** − **	** − **	** − **	Central	White & green	Pale green
*C. longa*	Me-1	Japan	Yellowish orange	Green	** − **	** − **	** − **	Central	**/**	**/**
*C. longa*	Q-62	Thailand	Bright yellow	Green	** − **	** − **	** − **	Central	White	Pale green
*Curcuma* sp. (*C. longa*)	94,009	Thailand	Orange	Green	** − **	** − **	** − **	**/**	**/**	**/**
*C. longa*	No-16	Indonesia	Orange-red	Green	** − **	** − **	** − **	Central	**/**	**/**
*C. longa*	Tsu-7	Indonesia	Orange-red	Green	** − **	** − **	** − **	Central	**/**	**/**
*Curcuma* sp. (*C. longa*)	97021	India	Bright yellow	Green	** − **	** − **	** − **	**/**	**/**	**/**
*C. longa*	92005	Nepal	Bright yellow	Green	** − **	** − **	** − **	**/**	**/**	**/**
*C. aromatica* (Jp)	01005	Japan	Light yellow	Green	** − **	** − **	** ++ **	**/**	**/**	**/**
*C. aromatica* (Jp)	10164	Japan	Light yellow	Green	** − **	** − **	** ++ **	**/**	Pink	Pale green
*C. aromatica* (Jp)	K-116	Japan	Light yellow	Green	** − **	** − **	** ++ **	Lateral	Pink	Pale green
*C. aromatica* (Jp)	H-21	Japan	Light yellow	Green	** − **	** − **	** ++ **	Lateral	Pink	Pale green
*C. aromatica* (Jp)	Me-3	Japan	Light yellow	Green	** − **	** − **	** ++ **	Lateral	Pink	Pale green
*C. aromatica* (Jp)	J-12	Japan	Light yellow	Green	** − **	** − **	** ++ **	Lateral	Pink	Pale green
*C. aromatica* (Cn)	Q-35	China	Light yellow	Green	** − **	** − **	** ++ **	Lateral	Pink	Pale green
*C. aromatica* (Cn)	Q-36	China	Light yellow	Green	** − **	** − **	** ++ **	Lateral	Pink	Pale green
*C. aromatica* (Cn)	Q-37	China	Light yellow	Green	** − **	** − **	** ++ **	Lateral	Pink	Pale green
*Curcuma* sp. (*C. aromatica*)	94006	Thailand	Light yellow	Green	** − **	** − **	** ++ **	**/**	**/**	**/**
*C. phaeocaulis*	Q-38	China	Greenish blue & yellow	Rusty	** + + **(wide)	** − **	** + **	Lateral	White & pink	Whilt green
*C. phaeocaulis*	Q-39	China	Greenish blue & yellow	Rusty	** + + **(wide)	** − **	** + **	Lateral	White & pink	Whilt green
*C. phaeocaulis*	Q-40	China	Greenish blue & yellow	Rusty	** + + **(wide)	** − **	** + **	Lateral	White & pink	Whilt green
*C. phaeocaulis*	Q-42	China	Greenish blue & yellow	Rusty	** + + **(wide)	** − **	** + **	Lateral	White & pink	Whilt green
*C. phaeocaulis*	Q-43	China	Greenish blue & yellow	Rusty	** + + **(wide)	** − **	** + **	Lateral	White & pink	Whilt green
*C. phaeocaulis*	Q-64	China	Greenish blue & yellow	Rusty	** + + **(wide)	** − **	** + **	Lateral	White & pink	Whilt green
*Curcuma* sp. (*C. phaeocaulis*)	94010	Thailand	Blue	Rusty	** + + **(wide)	** − **	** + **	**/**	**/**	**/**
*C. aeruginosa*	Q-41	China	Yellowish green & yellow	Rusty	** + **(narrow)	** − **	** − **	Central, lateral	White & pink	Whilt green
*C. aeruginosa*	Q-47	China	Yellowish green & yellow	Rusty	** + **(narrow)	** − **	** − **	Central	White & pink	Whilt green
*C. aeruginosa*	10167	Indonesia	Yellowish green & yellow	Rusty	** + **(on midvein)	** − **	** − **	Central, lateral	White & pink	White green
*C. zedoaria* (Jp)	91014	Japan	Pale blue	Green	** + **(on distal side)	** − **	** − **	**/**	**/**	**/**
*C. zedoaria* (Jp)	K-100	Japan	Purplish blue	Green	** + **(on distal side)	** − **	** − **	Lateral	Purple	Pale green
*C. zedoaria* (Jp)	Ko-93	Japan	Purplish blue	Green	** + **(on distal side)	** − **	** − **	Lateral	Purple	Pale green
*C. zanthorrhiza*	Q-48	China	Yellowish orange	Green	** + **(on midvein)	** − **	** − **	Lateral	Purple	Pale green
*C. zanthorrhiza*	K-105	Japan	Orange	Green	** + **(on midvein)	** − **	** − **	Lateral	Purple	Pale green
*C. zanthorhiza*	10163	Malaysia	Yellowish orange	Green	** + **(on midvein)	** − **	** − **	**/**	Purple	Pale green
*C. zanthorrhiza*	94020	Indonesia	Orange	Green	** + **(on midvein)	** − **	** − **	**/**	**/**	**/**
*C. wenyujin*	Q-49	China	Pale yellow	Green	** − **	** − **	** − **	Lateral	Pink	Green
*C. wenyujin*	Q-70	China	Pale yellow	Green	** − **	** − **	** − **	Lateral	Pink	Green
*C. kwangsiensis*	Q-63	China	Yellowish white	Green	** − **	** + **	** ++ **	Lateral	Pink	Green
*C. kwangsiensis*	Q-66	China	Yellowish white	Green	** − **	** + **	** ++ **	Central, lateral	Red	Green
*C. kwangsiensis*	Q-67	China	Yellowish white	Rusty	** + **(narrow)	** + **	** ++ **	Lateral	Red	Green
*C. kwangsiensis*	Q-68	China	Yellowish white	Green	** − **	** + **	** ++ **	Lateral	Pink	Green
*C. kwangsiensis*	Q-69	China	Yellowish white	Green	** − **	** + **	** ++ **	Central, lateral	Red	Green & red
*Curcuma sp.* (C. *amada*)	00591	India	Yellow	Green	** − **	** − **	** − **	**/**	**/**	**/**
*Curcuma sp.* (C. *mangga*)	00959	Thailand	Pale yellow	Green	** − **	** − **	** + **	**/**	**/**	**/**
*Curcuma* sp. (*C. petiolata*)	94008	Thailand	Pale yellow	Green (with white margin)	** − **	** − **	** − **	**/**	**/**	**/**
*C. sichuanensis*	Q-50	China	Light yellow	Green	** − **	** − **	** − **	Central	White & pink	Pale green

** + **presence, − absence, **/** data unavailable

*Scientific names of *C. aromatica* and *C. zedoaria* are followed by the name of each country where the plant was produced: (Jp), Japan; (Cn), China

**Fig. 1:**
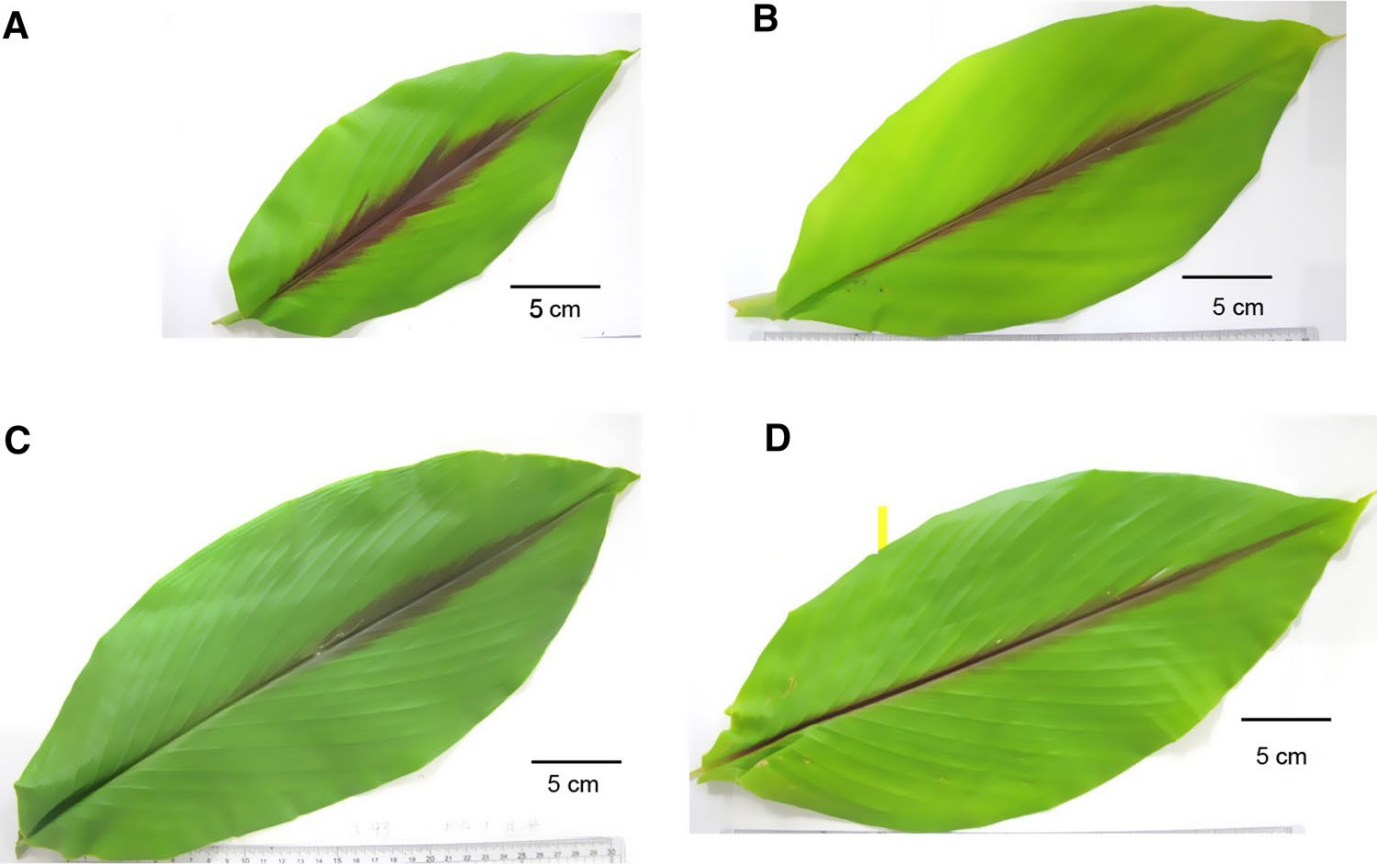
Leaves of *Curcuma* species specimens: **A** Q40, *C. phaeocaulis*; **B** Q41, *C. aeruginosa*; **C** Ko-93, *C. zedoaria* (Jp); and **D** Q48, *C. zanthorrhiza*

**Fig. 2:**
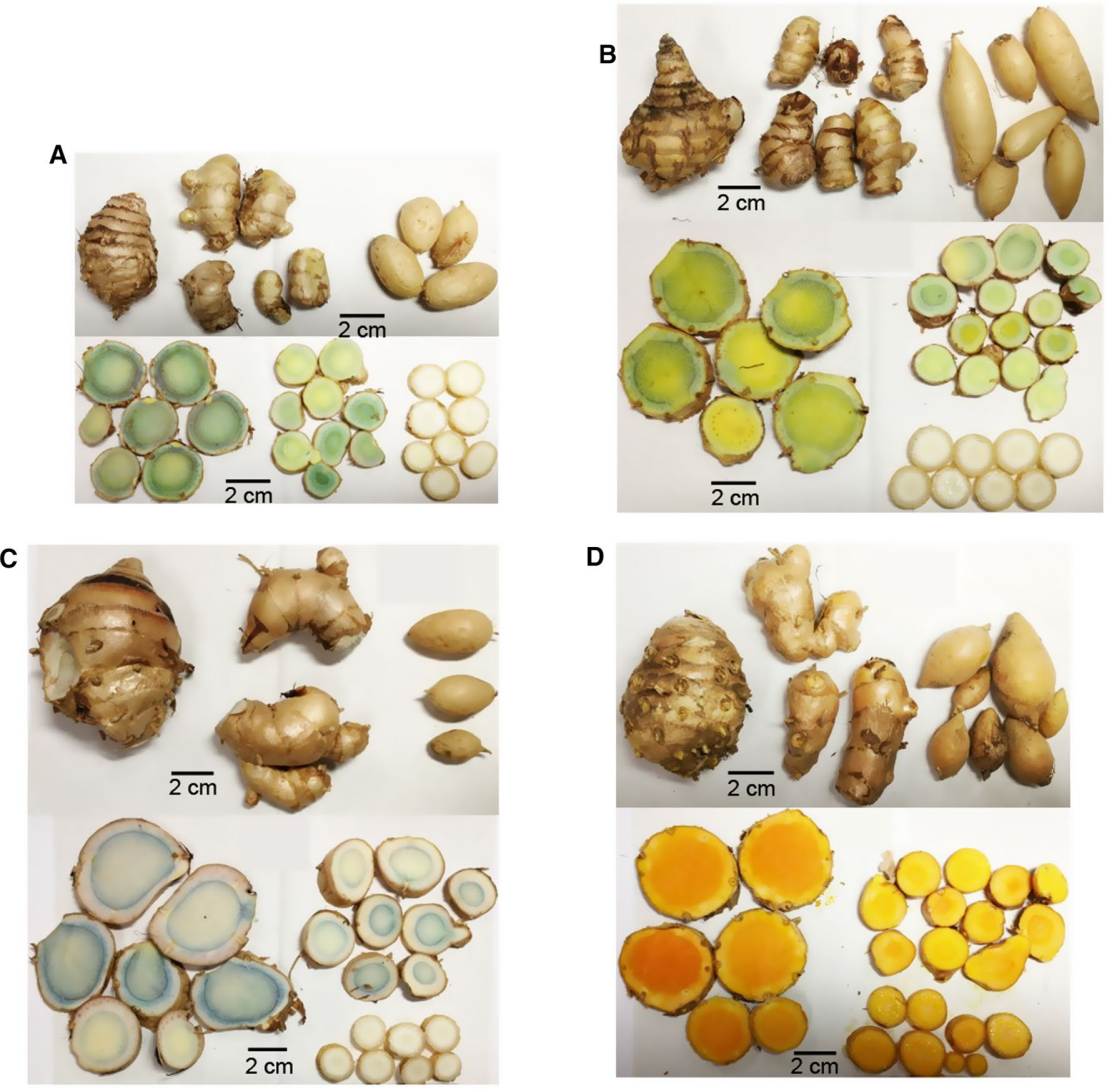
Rhizomes and tubers of *Curcuma* species specimens: **A** Q40, *C. phaeocaulis*; **B** Q41, *C. aeruginosa*; **C** Ko-93, *C. zedoaria* (Jp); and **D** Q48, *C. zanthorrhiza*

The *C. longa* specimens had green-colored and glabrous leaves, and a bulbed rhizome with several finger-shaped rhizomes attached whose cut surface was bright yellow to orange. Two uncertain specimens 94009 and 97021 which had the above characteristics were identified as *C. longa*. The inside color of *C. longa* rhizome varied depending on the producing areas; for instance, it was bright yellow in China and Japan, orange in Thailand and reddish orange in Indonesia. The *C. aromatica* from Japan and China was discriminated from *C. longa* by the features of leaves with a pubescent lower surface, light-yellow rhizomes as well as separately arising inflorescences. Uncertain specimen 94006 had similar morphology to Japanese *C. aromatica*. *C. phaeocaulis*, *C. aeruginosa*, *C. zedoaria* and *C. zanthorrhiza* had a purple band along the midrib on the upper leaf surface; however, similarities and variabilities in this feature made it difficult to identify these species, particularly for *C. phaeocaulis* and *C. aeruginosa* which also had similar rust-colored leaf sheaths. The Flora of China mentions that *C. phaeocaulis*, *C. aeruginosa,* and *C. zedoaria* have long been misidentified in China [20]. Based on comparative and precise observations, the morphological differences between *C. phaeocaulis* and *C. aeruginosa* were summarized: *C. phaeocaulis* had a wide purple band on the upper leaf surface (Fig. 1A) and short hairs on the lower surface, lateral inflorescence and inside color of rhizomes was greenish-blue and yellow (Fig. 2A); whereas *C. aeruginosa* had a narrow purple band on the upper leaf surface (Fig. 1B) and a glabrous lower surface, central or lateral inflorescence and the rhizome interior was yellowish-green and yellow (Fig. 2B). According to these morphological characters, uncertain specimen 94010 was identified as *C. phaeocaulis*, with rust-colored leaf sheaths, a wide purple band on upper leaf surfaces and blue rhizome interior. Three specimens of *C. zedoaria* from Japan were characterized by green leaf sheaths, a purple band along the midvein from the middle to the tip of leaves (Fig. 1C) and rhizomes with pale or purplish-blue interior (Fig. 2C). The *C. zanthorrhiza* specimens were similar to *C. zedoaria* cultivated in Japan but were discriminated by yellowish-orange to orange rhizomes (Fig. 2D) and an extremely narrow purple band along the leaf midrib (Fig. 1D). The *C. kwangsiensis* specimens were characterized by dense hairs on both leaf surfaces and yellowish white rhizomes, although there were some variations such as in leaf sheath color, presence or absence of a purple band on the leaf blade and inflorescence position as previously reported [25]. Two *Curcuma* specimens, one labeled *C. amada*, originally obtained from India and another labeled *C. mangga* from Thailand had mango-smelling rhizomes with yellow and pale yellow inside color, green leaf sheaths and green leaves; however, other morphological features were unavailable. The *C. petiolata* specimen had oblong to ovate leaves with long petioles and pale-yellow rhizomes. Leaves of specimen 94008 had creamy white margins similar to some varieties of *C. petiolata*. The *C. sichuanensis* specimen had similar features to *C. longa* except for a light-yellow rhizome interior.

### *trn*K intron sequences

The *trn*K intron sequences of all plant specimens and crude drug samples were successfully determined, except for plant specimens T004 and I-0007. In accord with our previous reports [14–16, 25], *C. longa* showed Ltk(11T) and Ltk(10T) types of sequences, and both *C. kwangsiensis* and *C. wenyujin* had a K(gl)Wtk type of sequence. In addition, *C. aromatica* (Jp), *C. zedoaria* (Jp) and *C. phaeocaulis* showed Atk, K(pl)Ztk and Ptk types of sequences, respectively (Table 4). In addition to these six types of recorded sequences, five new types were detected. Three new types showed high similarities to the type Ltk(11T) sequence, and were named Ltk(11T)in-1, Ltk(11T)in-2 and Ltk(11T)139A. The Ltk(11T)in-1 and -2 types had a 5-bp “CATAA” insertion either in front of or behind the sequence unit “TACAA” at the alignment positions 2433–2442; Ltk(11T)139A type had an adenine instead of guanine at position 139. The Ltk(11T)in-1 and -2 types were detected in two plant specimens of *C. longa* and two crude drug samples from Thailand. The Ltk(11T)139A type was detected in crude drug sample D8685 from Sri Lanka, which has been generally considered as *C. longa* [3]. The other two new types showed high similarities to the K(pl)Ztk type sequence but differed by the number of poly-adenine and poly-thymine observed from positions 205 and 502, respectively. The K(pl)Ztk type sequence detected in *C. zedoaria* from Japan had 6 adenines and 14 thymines at these two sites [renamed K(pl)Ztk(6A14T)], whereas new types had 7 adenines and 15 or 13 thymines [named K(pl)Ztk(7A15T) or K(pl)Ztk(7A13T), respectively]. The K(pl)Ztk(7A15T) sequence type was detected in five crude drug samples from Thailand. The K(pl)Ztk(7A13T) sequence type was detected in crude drug samples D21642, D24876 and D30519 from Thailand and crude drug sample D22836 from India.

**Table 4:**
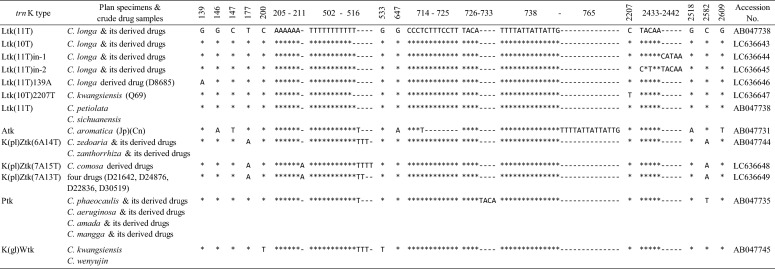
The *trn*K intron sequences detected in *Curcuma* plant specimens and crude drug samples

Numbers above sequence indicate the alignment positions. Hyphen (-) denotes alignment gap, asterisk (*) indicates nucleotide is same with that in the first sequence

There were four species for which the *trn*K intron sequences were analyzed for the first time: *C. petiolata* and *C. sichuanensis* had a Ltk(11T) sequence that was the same as for *C. longa*; *C. zanthorrhiza* had a K(pl)Ztk(6A14T) sequence that was the same as for *C. zedoaria* (Jp); and *C. aeruginosa*, *C. amada* and *C. mangga* had a Ptk sequence that was the same as for *C. phaeocaulis*. These species pairs as well as another pair, *C. wenyujin* and *C. kwangsiensis*, could not be discriminated using the *trn*K intron sequences. The *trn*K sequence types of crude drug samples are summarized in Table 2.

One *C. kwangsiensis* specimen (Q69) had a Ltk(10T)2207T type of sequence with a thymine instead of cytosine at position 2207 in Ltk(10T), but not a K(gl)Wtk type. As reported in our previous paper [25], *C. kwangsiensis* is probably of hybrid origin and several species including *C. wenyujin*, *C. phaeocaulis* and *C. longa* might be involved in its formation. The *trn*K intron sequence of Q69 suggested that this specimen might have a maternal line from *C. longa* because of the maternal heritance of the chloroplast DNA. The same phenomenon was detected in the crude drug sample “Kamin oi” from Thailand, which has been generally considered as the rhizome of *C. zedoaria*; samples D21642 and D24876 showed K(pl)Ztk(7A13T) type, and sample D24864 showed Ltk(11T) type, although these three samples had the same morphological features.

### ILP analysis of *DCS* introns I and II and *CURS* intron regions

**Fig. 3:**
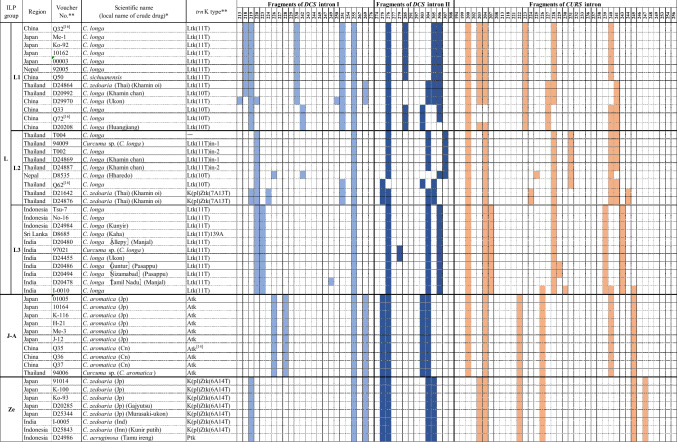

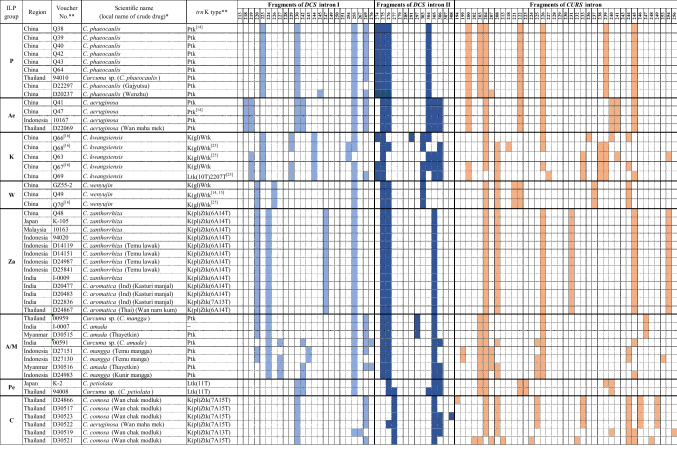
ILP patterns of all plant specimens and crude drug samples. Voucher no. preceded by “D” indicates a crude drug sample. *The scientific name of crude drug samples was deduced from the local name, shown in parentheses. **[16], samples whose *trn*K intron sequences and ILP patterns were previously reported in reference [16]; [14, 15, 25], samples whose *trn*K intron sequences were previously reported in references [14, 15, 25]

The three intron regions of 59 plant specimens and 41 crude drug samples were amplified successfully. The length of the amplified DNA fragments ranged from 213 to 276 bp in *DCS* intron I region, 274 to 308 bp in *DCS* intron II region and 194 to 256 bp in *CURS* intron region. The ILP patterns based on both number and length of the amplified fragments in the three intron regions are shown in Fig. 3. The ILP patterns revealed high intraspecies consistency in *C. aromatica* from Japan and China; *C. zedoaria* from Japan, India and Indonesia; and *C. phaeocaulis*, *C. aeruginosa*, *C. wenyujin* and *C. zanthorrhiza*; but showed intraspecies polymorphism in *C. longa*, *C. kwangsiensis*, *C. amada*, *C. mangga* and *C. comosa*.

**Fig. 4:**
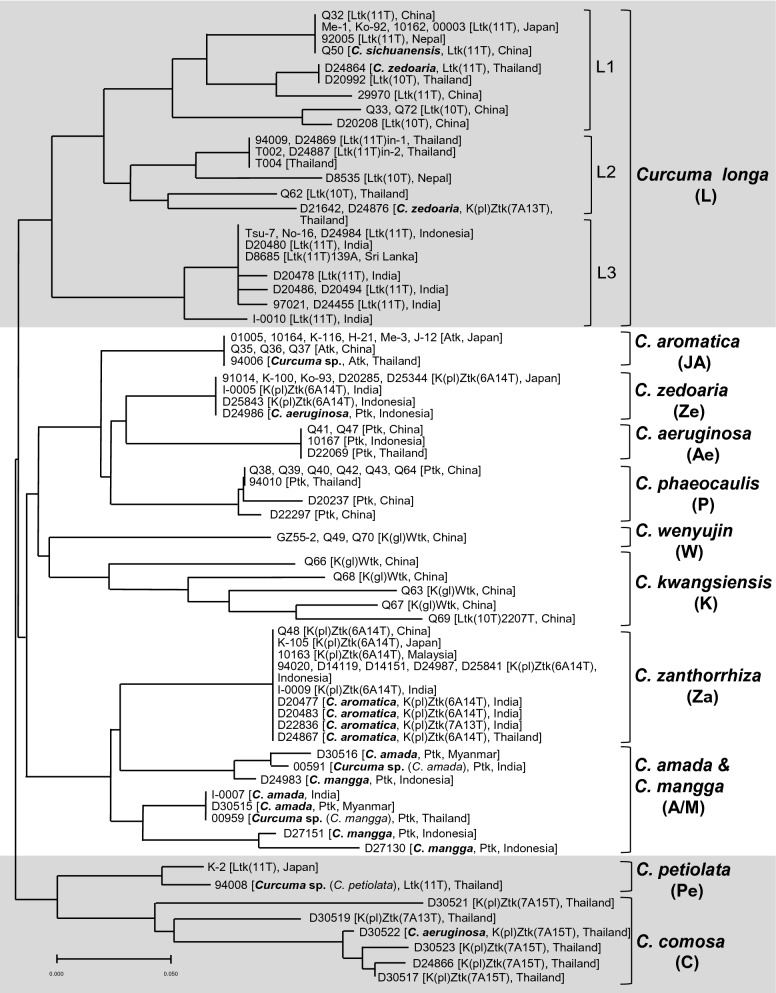
Dendrogram constructed by neighbor-joining method based on similarity of the ILP patterns. The scale under the tree indicates branch length

Based on similarities of the ILP patterns of all samples, an unrooted tree was constructed using the NJ method (Fig. 4). In the NJ tree, *C. petiolata* and *C. comosa* formed a clade, separated from the large clade comprising the rest of the species. The large clade was further divided into two subclades, in which all the plant specimens and crude drug samples of *C. longa* formed one subclade, and another subclade comprising the other species was further divided into two branches: one composed of *C. aromatica*, *C. zedoaria*, *C. aeruginosa*, *C. phaeocaulis*, *C. wenyujin,* and *C. kwangsiensis*; and the other composed of *C. zanthorrhiza*, *C. amada* and *C. mangga*. The similarity of ILP patterns in the specimens and samples of the respective species led to clear clustering in the NJ tree, with 11 main groups corresponding to the respective species: group **L** (*C. longa*), group **JA** (Japanese population of *C. aromatica*), group **Ze** (*C. zedoaria*), group **A**e (*C. aeruginosa*), group **P** (*C. phaeocaulis*), group **W** (*C. wenyujin*), group **K** (*C. kwangsiensis*), group **Za** (*C. zanthorrhiza*), group **A/M** (*C. amada* or *C. mangga*), group **Pe** (*C. petiolata*) and group **C** (*C. comosa*).

Group **L** (*C. longa*) included all the plant specimens or crude drug samples of *C. longa* that were introduced from or produced in Japan, China, Thailand, Indonesia, India, Sri Lanka and Nepal, as well as plant specimen Q50 of *C. sichuanensis* from China and crude drug samples D24864, D21642 and D24876 from Thailand (Fig. 3). This group was further divided into three subgroups and this grouping was highly consistent with the geographical origins of the included samples. Therefore, they were tentatively assigned as China–Japan (**L1**), Thailand (**L2**) and India–Indonesia (**L3**) subgroups. In subgroup **L1**, six plant specimens of *C. longa* from Japan, China, and Nepal showed identical ILP patterns as well as *trn*K intron sequences, and plant specimen Q50 of *C. sichuanensis* also showed the same ILP pattern and the same Ltk(11T) type of *trn*K intron sequence as *C. longa*. According to the Flora of China [20], *C. sichuanensis* has strong morphological similarities to *C. longa*, only differing from *C. longa* by light inside color of rhizomes and coma bracts as well as a yellow band in the labellum. Xiao et al. suggested that *C. sichuanensis* should be treated as *C. longa* cv. *sichuanensis* or *C. longa* complex based on their field investigation in Sichuan, China [17]; the morphological and molecular evidence obtained in our study supports this proposal. Crude drug sample D24864 collected from Thailand was included in this subgroup. Although this sample was deduced to be *C. zedoaria* from its local name “Khamin oi,” the ILP pattern and Ltk(11T) type of *trn*K intron sequence of this sample suggested its botanic source might be a strain closely related to *C. longa*. In subgroup **L2**, three plant specimens and two crude drug samples of *C. longa* from Thailand showed identical ILP patterns (Fig. 3). Two crude drug samples D21642 and D24876 with the local name “Khamin oi” had the same ILP pattern, which somewhat differed from that of *C. longa* from Thailand, and their K(pl)Ztk(7A13T) type of *trn*K intron sequence differed from both that of *C. longa* and *C. zedoaria*. The above results suggest that these two “Khamin oi” samples might be of hybrid origin in which *C. longa* and species with a K(pl)Ztk(7A13T) type of *trn*K intron sequence were involved in hybridization. Further study is needed to clarify the botanical origin of “Khamin oi” and to investigate the variability of ILP pattern in hybrid plants. The subgroup **L3** included seven plant specimens or crude drug samples from India, three plant specimens or crude drug samples from Indonesia and one crude drug sample from Sri Lanka. Except for six samples from India, the samples showed an identical ILP pattern. Five ILP patterns were detected in the seven Indian specimens and samples, indicating considerable genetic polymorphism of *C. longa* in India. In fact, *C. longa* is widely cultivated in India and a number of cultivars have been developed to facilitate cultivation in various locations [3, 4]. In this study, four crude drug samples derived from four cultivars showed different ILP patterns.

The group **JA** included ten plant specimens of *C. aromatica*, including six specimens cultivated in Japan, three introduced from China and one uncertain specimen from Thailand. In Japan, *C. aromatica* has long been widely cultivated; however, the original source remains unclear. Japanese *C. aromatica* showed the same ILP pattern and *trn*K intron sequences as the three *C. aromatica* specimens introduced from China and one specimen introduced from Thailand (Fig. 3), as well as *C. chuanhuangjiang* from China [16]. Unfortunately, the type specimen of *C. aromatica* was unavailable and it has been pointed out that the name of *C. aromatica* is applied to several taxa in Asia that possess similar morphological features such as pale brown and aromatic rhizomes, entirely green leaves with a glabrous upper surface and pubescent lower surface, and lateral inflorescences [3, 26, 27]. In this study, the crude drug samples from India (D20477, D20483 and D22836) and Thailand (D24867), deduced to be *C. aromatica*, genetically differed from the Japanese and Chinese *C. aromatica* (Fig. 3) and so partly reflected such a situation.

The group **Ze** included four plant specimens of *C. zedoaria* (three cultivated in Japan and one introduced from India) as well as four crude drug samples produced in Japan or Indonesia. These samples showed an identical ILP pattern and the same K(pl)Ztk(6A14T) type of *trn*K intron sequence, except one crude drug sample (D24986, generally considered to be *C. aeruginosa* according to its local name “Temu ireng”) which showed a Ptk type of *trn*K intron sequence (Fig. 3). Taxonomically, *C. zedoaria* is quite similar to *C. aromatica*, due to historical nomenclatural confusion [28]. The name *C. zedoaria* has been applied to several taxa in Asia that possess a purple band along the midvein on the upper surface of leaves [3, 19, 21]. The Japanese population of *C. zedoaria* has been cultivated as “Gajyutsu” (“Ezhu” in Chinese) and has been prescribed in the Japanese Pharmacopoeia since the third edition in the year 1906 [29]; however, the original source of Japanese *C. zedoaria* is unclear. Kitamura et al. reported that “Gajyutsu” cultivated in Yakushima Island, Japan, was more similar to *C. aeruginosa* than *C. zedoaria* from Java, Indonesia, based on *trn*K intron sequence, random amplified polymorphic DNA analysis and essential oil composition [30]. Our study based on morphology, ILP markers and *trn*K intron sequence clearly distinguished Japanese *C. zedoaria* from Indonesian *C. aeruginosa*. However, crude drug sample D25843 from Indonesia deduced to be *C. zedoaria* through its local name “Kunir putih,” showed the same ILP pattern as well as *trn*K intron sequence as the Japanese *C. zedoaria*, which suggested that Japanese *C. zedoaria* had a close relation to Indonesian *C. zedoaria*. However, Indonesian crude drug sample D24986 showed identical ILP patterns to Japanese *C. zedoaria* but the same *trn*K intron sequence as *C. aeruginosa*, suggesting possible hybridization between these two species in Indonesia. Plant specimen I-0005 from India identified as *C. zedoaria* also showed the same ILP pattern and *trn*K intron sequence as Japanese *C. zedoaria*, suggesting close relations of the *C. zedoaria* populations in Japan, Indonesia and India. However, a further study based on morphological and molecular analyses of widely collected specimens from India and Indonesia is needed to determine any relationship between Japanese *C. zedoaria* and *C. zedoaria* in India and Indonesia.

The group **P**, including six plant specimens and two crude drug samples of *C. phaeocaulis* from China, showed the Ptk type of *trn*K intron sequence. All plant specimens from China and one specimen identified as *C. phaeocaulis* from Thailand showed an identical ILP pattern, whereas the two crude drug samples showed minor differences from this.

The group **Ae** included plant specimens Q41 and Q47 originally introduced from China and 10167 of *C. aeruginosa* introduced from Indonesia, as well as crude drug sample D22069 from Thailand, which was deduced to be *C. aeruginosa* from its local name “Wan maha mek.” All of them had an identical ILP pattern. Although *C. aeruginosa* showed the same Ptk type of *trn*K intron sequence as *C. phaeocaulis*, the distinguishable ILP patterns allowed clear discrimination of these two species.

The group **K** included five plant specimens of *C. kwangsiensis* introduced from China, among which four had K(gl)Wtk type and one had Ltk(10T)2207T type of *trn*K intron sequence. Our previous study using field investigation and morphological, genetic, and chemical analyses suggested *C. kwangsiensis* was of hybrid origin [25]. The variant ILP patterns of all five *C. kwangsiensis* specimens also indicated its genetic diversity.

In group **W**, three plant specimens of *C. wenyujin* introduced from China had identical ILP patterns and the same K(gl)Wtk type of *trn*K intron sequence.

The group **Za** was composed of five plant specimens of *C. zanthorrhiza* from China, Japan, Malaysia, Indonesia and India, and eight crude drug samples produced in Indonesia, India and Thailand. These samples had an identical ILP pattern and the same K(pl)Ztk(6A14T) type of *trn*K intron sequence (Fig. 3). Among the eight crude drug samples, four samples with local name “Temu lawak” from Indonesia were deduced to be *C. zanthorrhiza*, which was supported by our genetic analysis data. The other three samples from India (D20477, D20483 and D22836), deduced to be *C. aromatica* through the local name “Kasturi manjal,” were included in the **Za** group. *C. zanthorrhiza* is indigenous to South India; however, it has long been misidentified as *C. aromatica* in India [21]. Our molecular analysis revealed that the botanic source of the crude drug “Kasturi manjal” in India was *C. zanthorrhiza* not *C. aromatica*. A similar situation applied to crude drug sample D24867 from Thailand; it was deduced to be *C. aromatica* due to its local name “Wan narn kum,” while its botanic source was *C. zanthorrhiza*.

The group **A/M** included three plant specimens and five crude drug samples from Indonesia, Thailand, Myanmar and India. Plant specimen 00959 of *C. mangga* from Thailand, plant specimen I-0007 of *C. amada* from India and crude drug sample D30515 from Myanmar had an identical ILP pattern. In Bangladesh, *C. amada* was first recorded in the 1810s and its fresh rhizome with a smell of green mango is a distinguishable character of this species [31]. In the 1910s, another *Curcuma* species with rhizomes smelling of green mango was recorded in Java, Indonesia, and named *C. mangga*. Although the main morphological difference between the two species has been reported as a central inflorescence in *C. amada* and a lateral inflorescence in *C. mangga* [32], some reports described that *C. amada* in India can have a lateral or central inflorescence [33, 34]. Our molecular analysis revealed that their ILP patterns were indistinguishable. In addition, plant specimen 00591 of *C. amada* from India and crude drug samples D30516 of *C. amada* from Myanmar and D24983 of *C. mangga* from Indonesia belonged to the same subclade with *C. zanthorrhiza*, group **Za** in the phylogenetic tree (Fig. 4)*.*

The group **Pe** included one plant specimen of *C. petiolata* and uncertain plant specimen 94008. The two specimens showed Ltk(11T) type of *trn*K intron sequences, while specimen 94008 had an identical pattern in *DCS* intron I region and similar patterns in *DCS* intron II and *CURS* intron regions with the *C. petiolata* specimen. As described in the section “Morphological identification of plant specimens”, the specimen 94008 had characteristic leaves with a creamy white margin, similar to varieties of *C. petiolata*. Together with the molecular data, it is reasonable to conclude that this specimen was *C. petiolata*.

The group **C** included six crude drug samples from Thailand. In Thailand, the crude drug with the local name “Wan chak modluk” is generally considered to be *C. comosa*. The five “Wan chak modluk” samples showed different ILP patterns, among which four samples showed K(pl)Ztk(7A15T) type and one sample had the K(pl)Ztk(7A13T) type of *trn*K intron sequence. Crude drug sample D30522 with the name “Wan maha mek,” deduced to be *C. aeruginosa*, showed the same type of *trn*K intron sequence and similar ILP patterns to *C. comosa*. Therefore, we suspect the name of this sample is wrong.

The molecular information provided by ILP markers and *trn*K intron sequences was demonstrated to be useful for taxonomic arrangement of Asian *Curcuma* species and standardization of Asian *Curcuma* drugs. For obtaining more concise results on these difficult questions, however, further study including morphological comparison with the specimens from type locality and molecular investigation on variability of ILP pattern in hybrid plants is needed. Based on the present study, the botanical origins of “Khamin oi” and “Wan narn kum” from Thailand and “Kasturi manjal” from India are completely different from the general claims, suggesting these crude drugs should be used with caution.

## Conclusion

In this study, to elucidate specific molecular markers of medicinally used *Curcuma* species in Asia, to solve confusion on the reported botanical origin of crude drugs and to locate the original habitats of *C. aromatica* and *C. zedoaria* cultivated in Japan, molecular analysis based on the ILP markers in *DCS* and *CURS* genes and the *trn*K intron sequences was performed using 59 plant specimens and 42 crude drug samples, which belonged to 13 *Curcuma* species obtained from Asian countries. The ILP patterns of the respective species revealed high consistency in *C. aromatica*, *C. zedoaria*, *C. phaeocaulis*, *C. aeruginosa*, *C. wenyujin* and *C. zanthorrhiza*, and showed intraspecies polymorphism in *C. longa*, *C. kwangsiensis*, *C. amada*, *C. mangga* and *C. comosa*. The *C. longa* specimens and samples were separated into three subgroups which were highly consistent with their geographical origins. Based on the ILP markers and the *trn*K intron sequences, the botanical origins of “Khamin oi” were correctly determined to be *C. longa* or a hybrid between *C. longa* and other species with a K(pl)Ztk(7A13T) type of *trn*K intron sequence, and “Wan narn kum” from Thailand and “Kasturi manjal” from India were correctly determined to be *C. zanthorrhiza*. Moreover, morphological and molecular data showed that *C. aromatica* and *C. zedoaria* cultivated in Japan had close relations with *C. aromatica* from China and Thailand, and *C. zedoaria* from Indonesia and India, respectively. Thus, ILP markers in *DCS* and *CURS* genes combined with the *trn*K intron sequences were demonstrated to be useful for the standardization of Asian *Curcuma* drugs.

## Supplementary Information

Below is the link to the electronic supplementary material.

Supplementary file1 (PPTX 52 KB)
