# Correction to: Discrimination of *Curcuma* species from Asia using intron length polymorphism markers in genes encoding diketide-CoA synthase and curcumin synthase

**DOI:** 10.1007/s11418-023-01694-x

**Published:** 2023-03-29

**Authors:** Qundong Liu, Shu Zhu, Shigeki Hayashi, Osamu Iida, Akihito Takano, Katsunori Miyake, Suchada Sukrong, Mangestuti Agil, Indira Balachandran, Norio Nakamura, Nobuo Kawahara, Katsuko Komatsu

**Affiliations:** 1 Institute of Natural Medicine, University of Toyama, 2630, Sugitani, Toyama 930-0194 Japan; 2 Research Center for Medicinal Plant Resources, National Institutes of Biomedical Innovation, Health and Nutrition, Kumage-Gun, 17007-2 Nakatane-cho, Kagoshima, 891-3604 Japan; 3 Showa Pharmaceutical University, 3-3165 Higashi-Tamagawagakuen, Machidashi, Tokyo 194-8543 Japan; 4 Tokyo University of Pharmacy and Life Sciences, 1432-1 Horinouchi, Hachioji, Tokyo 192-0392 Japan; 5 Chulalongkorn University, 254 Phayathai Rd, Wang Mai, Pathum Wan District, Bangkok, 10330 Thailand; 6 Airlangga University, Jl. Airlangga No.4 - 6, Airlangga, Kec. Gubeng, Kota SBY, Jawa Timur 60115 Indonesia; 7 Center for Medicinal Plants Research, Arya Vaidya Sala, Kottakkal, Malappuram, Kerala 676503 India; 8 Doshisha Women’s College of Liberal Arts, Kodo, Kyotanabe City, Kyoto 610-0395 Japan

**Correction to: Journal of Natural Medicines (2022) 76:69–86** https://doi.org/10.1007/s11418-021-01558-2

The article Discrimination of *Curcuma* species from Asia using intron length polymorphism markers in genes encoding diketide-CoA synthase and curcumin synthase, written by Qundong Liu, Shu Zhu, Shigeki Hayashi, Osamu Iida, Akihito Takano, Katsunori Miyake, Suchada Sukrong, Mangestuti Agil, Indira Balachandran, Norio Nakamura, Nobuo Kawahara, Katsuko Komatsu, was originally published Online First without Open Access. After publication in volume 76, issue 1, page 69–86 the author decided to opt for Open Choice and to make the article an Open Access publication. Therefore, the copyright of the article has been changed to © The Author(s) 2023 and the article is forthwith distributed under the terms of the Creative Commons Attribution 4.0 International License, which permits use, sharing, adaptation, distribution and reproduction in any medium or format, as long as you give appropriate credit to the original author(s) and the source, provide a link to the Creative Commons licence, and indicate if changes were made. The images or other third party material in this article are included in the article’s Creative Commons licence, unless indicated otherwise in a credit line to the material. If material is not included in the article’s Creative Commons licence and your intended use is not permitted by statutory regulation or exceeds the permitted use, you will need to obtain permission directly from the copyright holder. To view a copy of this licence, visit http://creativecommons.org/licenses/by/4.0/.

The original article has been updated.

