# Supplementary material for: Discrimination of Curcuma species from Asia using intron length polymorphism markers in genes encoding diketide-CoA synthase and curcumin synthase
Source: J Nat Med. 2021 Sep 5;76(1):69–86. doi: 10.1007/s11418-021-01558-2 (PMC10050018; doi:10.1007/s11418-021-01558-2)
Supplement: Supplementary file 1 — Supplementary file1 (PPTX 52 KB) [file 11418_2021_1558_MOESM1_ESM.pptx]

## Slide 1
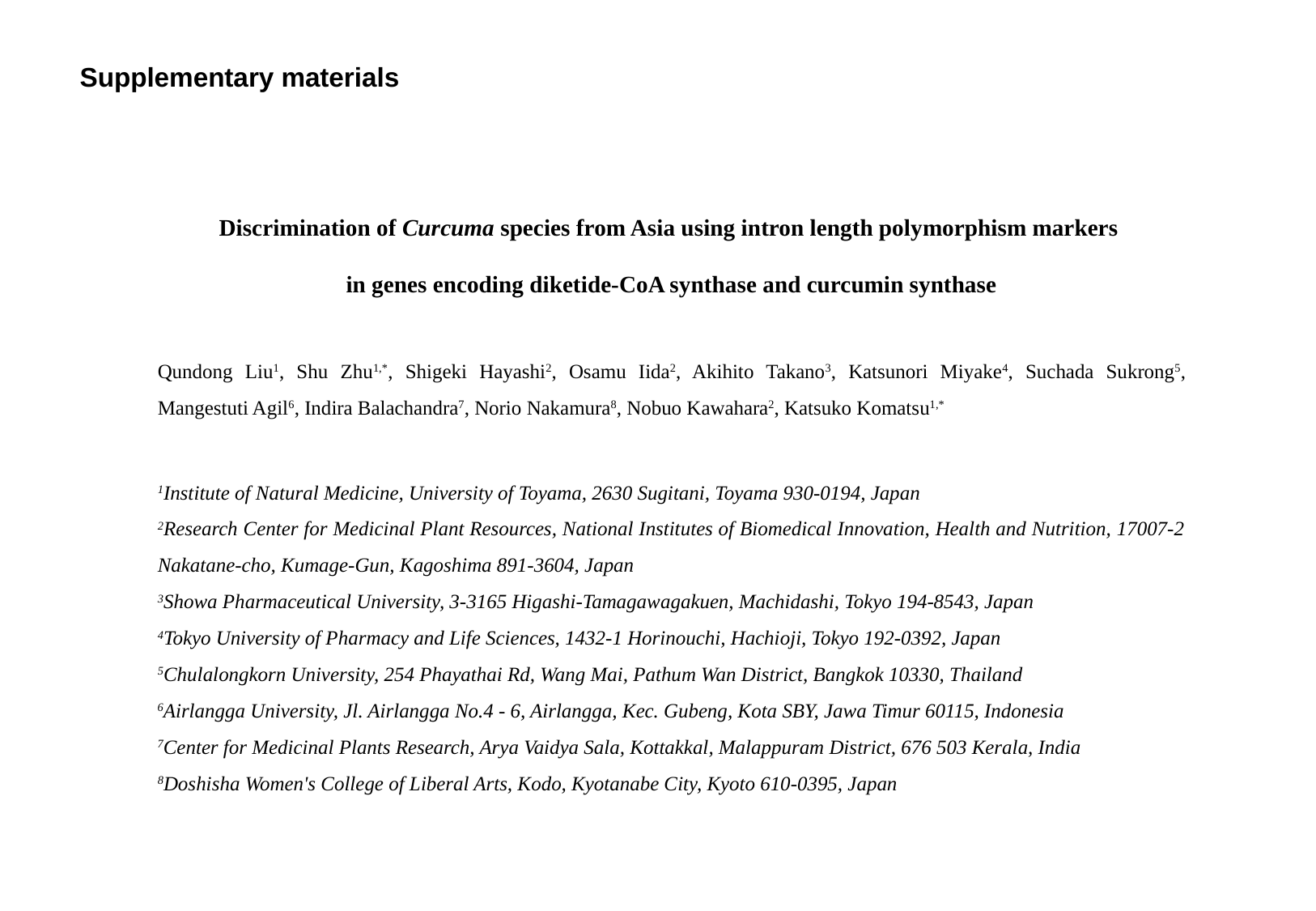

Supplementary materials
Discrimination of Curcuma species from Asia using intron length polymorphism markers
in genes encoding diketide-CoA synthase and curcumin synthase
Qundong Liu1, Shu Zhu1,*, Shigeki Hayashi2, Osamu Iida2, Akihito Takano3, Katsunori Miyake4, Suchada Sukrong5, Mangestuti Agil6, Indira Balachandra7, Norio Nakamura8, Nobuo Kawahara2, Katsuko Komatsu1,*
1Institute of Natural Medicine, University of Toyama, 2630 Sugitani, Toyama 930-0194, Japan
2Research Center for Medicinal Plant Resources, National Institutes of Biomedical Innovation, Health and Nutrition, 17007-2 Nakatane-cho, Kumage-Gun, Kagoshima 891-3604, Japan
3Showa Pharmaceutical University, 3-3165 Higashi-Tamagawagakuen, Machidashi, Tokyo 194-8543, Japan
4Tokyo University of Pharmacy and Life Sciences, 1432-1 Horinouchi, Hachioji, Tokyo 192-0392, Japan
5Chulalongkorn University, 254 Phayathai Rd, Wang Mai, Pathum Wan District, Bangkok 10330, Thailand
6Airlangga University, Jl. Airlangga No.4 - 6, Airlangga, Kec. Gubeng, Kota SBY, Jawa Timur 60115, Indonesia
7Center for Medicinal Plants Research, Arya Vaidya Sala, Kottakkal, Malappuram District, 676 503 Kerala, India
8Doshisha Women's College of Liberal Arts, Kodo, Kyotanabe City, Kyoto 610-0395, Japan

## Slide 2
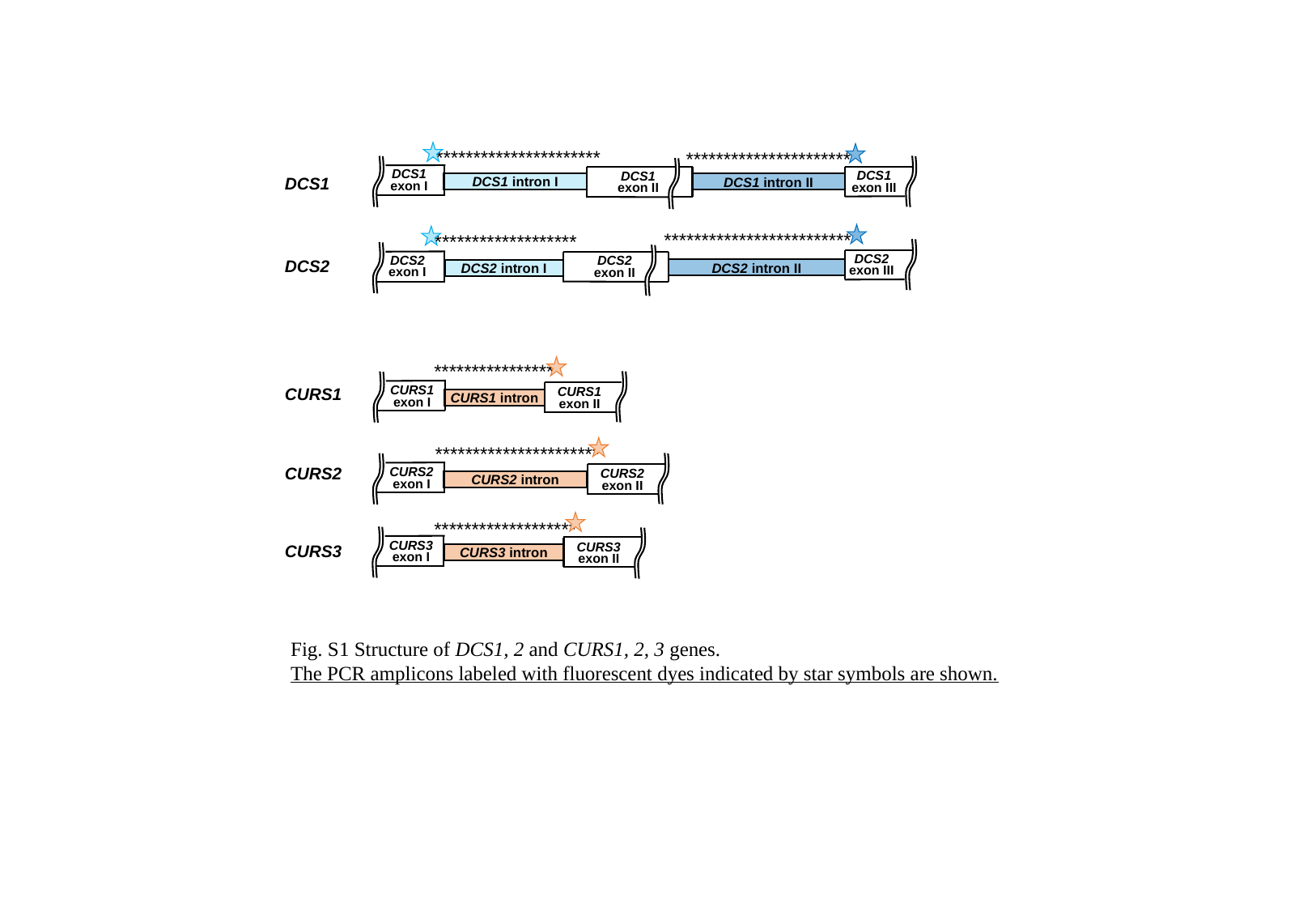

***********************
**********************
DCS1 exon I
DCS1 exon III
DCS1 exon II
DCS1
DCS1 intron I
DCS1 intron II
*************************
********************
DCS2 exon III
DCS2 exon I
DCS2 exon II
DCS2
DCS2 intron II
DCS2 intron I
****************
CURS1 exon I
CURS1
CURS1 exon II
CURS1 intron
**********************
CURS2
CURS2 exon I
CURS2 exon II
CURS2 intron
*******************
CURS3 exon I
CURS3 exon II
CURS3
CURS3 intron
Fig. S1 Structure of DCS1, 2 and CURS1, 2, 3 genes.
The PCR amplicons labeled with fluorescent dyes indicated by star symbols are shown.

## Slide 3
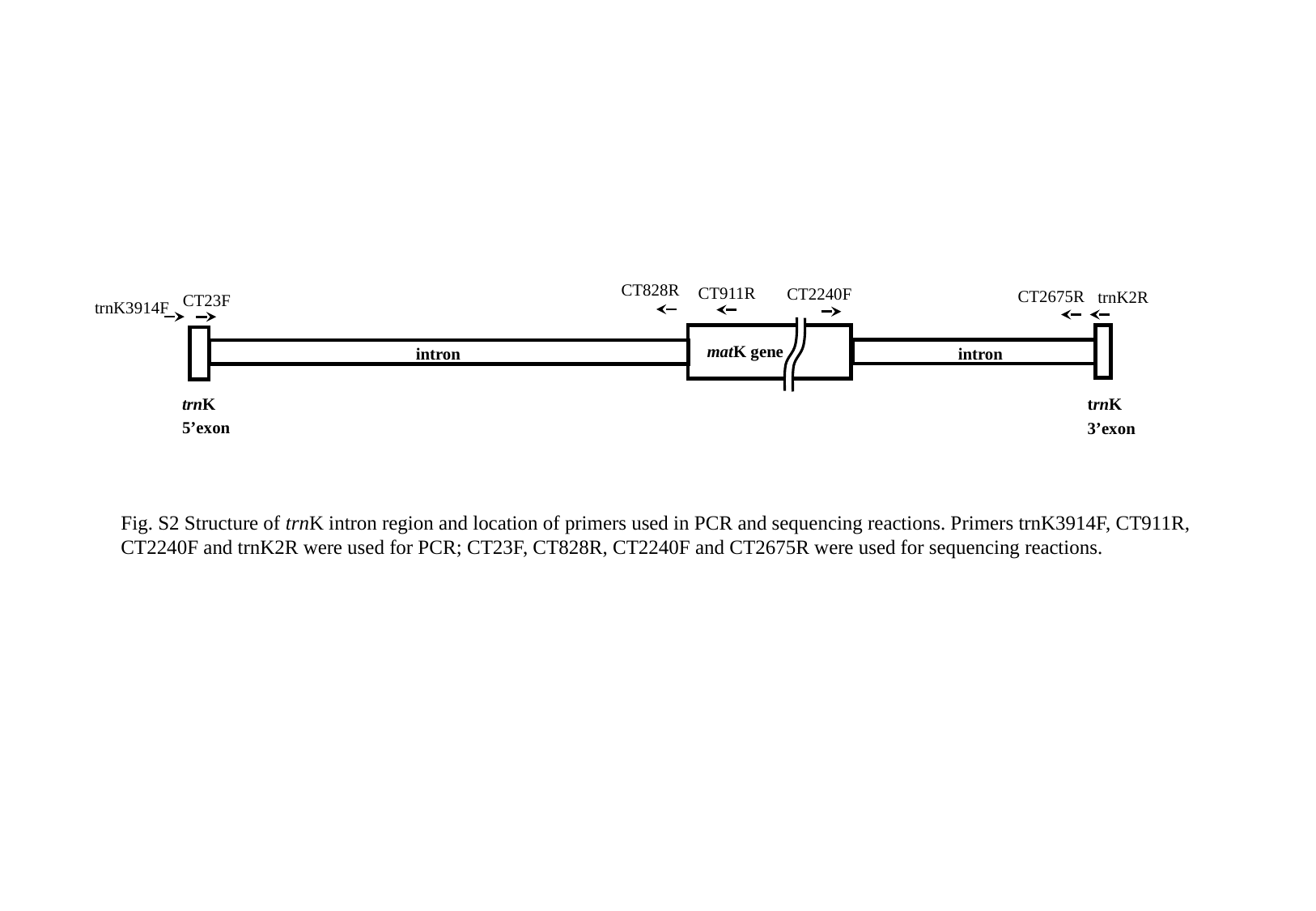

CT828R
CT911R
CT2240F
CT2675R
trnK2R
CT23F
trnK3914F
intron
intron
matK gene
trnK 5’exon
trnK
3’exon
Fig. S2 Structure of trnK intron region and location of primers used in PCR and sequencing reactions. Primers trnK3914F, CT911R, CT2240F and trnK2R were used for PCR; CT23F, CT828R, CT2240F and CT2675R were used for sequencing reactions.
